# Meta-Analysis of the Prognostic Value of Smad4 Immunohistochemistry in Various Cancers

**DOI:** 10.1371/journal.pone.0110182

**Published:** 2014-10-15

**Authors:** Yiping Du, Xin Zhou, Zebo Huang, Tianzhu Qiu, Jian Wang, Wei Zhu, Tongshan Wang, Ping Liu

**Affiliations:** 1 Department of Oncology, First Affiliated Hospital of Nanjing Medical University, Nanjing, China; 2 Cancer Center of Nanjing Medical University, Nanjing, China; Innsbruck Medical University, Austria

## Abstract

**Background:**

Accumulating evidence indicates that Smad4 (DPC4) plays a fundamental role in the development and prognosis of several types of cancer. The objective of this study was to conduct a meta-analysis to evaluate whether the loss of Smad4 staining could serve as a prognostic marker.

**Methods:**

A comprehensive meta-analysis was conducted using major useful databases to determine the relationship between the immunohistochemical detection of Smad4 and the survival of patients with various cancers. We used hazard ratios (HRs) with 95% confidence interval (CIs) as the effect estimation to evaluate the association of Smad4 with overall survival (OS), cancer-specific survival (CSS) or recurrence-free survival (RFS). The relationship between the clinical characteristics of patients and Smad4 was also evaluated using the odds ratio (OR).

**Results:**

A total of 7570 patients from 26 studies were included in the analysis. The pooled results showed that loss of Smad4 staining was a negative predictor of OS with an HR of 1.97 (95% CI: 1.55–2.51; P_heterogeneity_<0.001) and CSS/RFS (HR = 1.81; 95% CI: 1.30–2.54; P_heterogeneity_<0.001). In addition, loss of Smad4 staining was more likely to be found in older (OR = 1.69, 95% CI: 1.09–2.61; P_heterogeneity_ = 0.648) colorectal cancer patients with a late tumor stage (OR = 2.31, 95% CI: 1.71–3.10; P_heterogeneity_ = 0.218) and in gastric cancer patients with lymph node metastasis (OR = 2.11, 95% CI: 1.03–4.34; P_heterogeneity_ = 0.038).

**Conclusion:**

Based on these results, our meta-analysis provided evidence that loss of Smad4 staining could act as an unfavorable biomarker in the prognosis of various cancers and should be used as a powerful tool in future clinical trials.

## Introduction

For many years, cancer has been the leading cause of death worldwide, making it a global health problem [1]. Newer diagnostic methods to detect cancer with improved sensitivity and specificity have been developed. However, because cancer is characterized by invasion and metastasis, which are the main factors contributing to its high mortality rate, the prognosis of cancer remains poor, with a disappointing five-year survival rate. Digestive system tumors, particularly gastric, colorectal and pancreatic cancer, are common malignancies and are the leading cause of cancer-related deaths worldwide. Clinico-pathological characteristics—for example, tumor size and stage—do not fully predict individual clinical outcomes. Thus, molecular prognostic biomarkers to predict the progression of the disease, response to treatment, and survival were widely explored during the past decades.

Recently, the presence of certain proteins, such as matrix metalloproteinase 9 (MMP-9), C-reactive protein and vascular endothelial growth factor (VEGF), has been found to be associated with a poor prognosis in multiple cancers [2]–[4]. Among these biomarkers, Smad4 has attracted increasing research attention. Smad4, also termed DPC4, is a tumor suppressor gene that is recognized as a common intracellular mediator that can alter transforming growth factor β (TGF-β) signaling to promote tumor progression. Smad4-dependent TGF-β signaling is common during tumor development and progression and can modulate cell proliferation, affect cell motility, regulate the epithelial-mesenchymal-transition (EMT) process and affect sensitivity to clinical therapy [5]–[7]. Inactivation of the Smad4-induced deregulation of the TGF-β superfamily signaling is well established in some cancers. Moreover, it was found that Smad4 was associated with tumor invasion, metastasis and prognosis in different cancers [8]–[10]. However, the potential prognostic value of the immunohistochemical detection of Smad4 in various types of cancer is inconsistent. For instance, in pancreatic cancer, Milind Javle et al. [11] reported that the expression level of Smad4 was not associated with OS. However, it was found that the immunohistochemical detection of Smad4 was an independent and significant prognostic factor for overall and disease-free survival in the study of Hua et al. [12]. Regarding colorectal and gastric cancer, inconsistent results concerning the prognostic value of Smad4 were also found in different articles. Thus, an effective meta-analysis to explore the prognostic value of Smad4 in various cancers is urgently needed. To our knowledge, this study is the first comprehensive meta-analysis to explore the prognostic role of Smad4 in different types of cancer.

## Materials and Methods

### Publication search and inclusion criteria

PubMed, EMBASE and ISI Web of Science were searched to collect potentially relevant published studies. Medical subheading (MeSH) terms related to Smad4 (or DPC4) in combination with words related to cancer (or tumor or neoplasms or carcinoma) and terms related to prognosis (or outcome or survival or prognostic) were used to retrieve eligible studies through February 2014. The article references and review articles were also examined to identify additional potentially relevant studies. Studies were considered eligible if they met the following criteria: (a) included cancer patients who were pathologically confirmed; (b) investigated the association between the immunohistochemical detection of Smad4 and overall survival (OS) or cancer-specific survival (CSS) or recurrence-free survival (RFS); and (c) were published as a full paper in English. Studies were excluded based on the following criteria: (a) letters, reviews, case reports or laboratory studies; (b) studies with duplicate data or a repeated analysis; (c) lack of key information for further analysis; and (d) non-human research.

### Data extraction

Data were evaluated and extracted independently by two investigators under the guidelines of the Dutch Cochrane Centre proposed by the Meta-analysis of Observational Studies in Epidemiology (MOOSE) [13]. For each study, the following information was recorded: first author, year of publication, country of origin, ethnicity, total number of cases, cancer type, stage, follow ups and hazard ratios (HRs) with their 95% confidence intervals (CIs) and *P* value. For discrepancies, a consensus was reached on each item among the authors.

### Statistical analysis

HRs with their 95% CIs obtained from studies were used to calculate pooled HRs. When the data were not directly reported, a mathematical estimation was performed by calculating the necessary data according to the methods published by Parmer et al. [14]. We investigated the heterogeneity of pooled results using Cochran's Q test and the Higgins I-squared statistic. If the result of the *Q* test revealed *P*_heterogeneity_>0.1 and *I^2^*<50%, indicating the absence of heterogeneity, then a fixed-effects model (the Mantel–Haenszel method) was used to estimate the summary HRs/ORs [15]. Otherwise, the random-effects model (the DerSimonian and Laird method) was used [16]. Stratification and meta-regression analyses were used to detect the potential heterogeneity among studies. Begg's funnel plot and Egger's linear regression test were conducted to examine publication bias in the literature, and p<0.05 was considered significant. All statistical analyses were performed using the STATA software, version 12.0 (STATA Corporation, College Station, TX, USA). All P values were two-sided.

## Results

### Study characteristics

**Figure 1:**
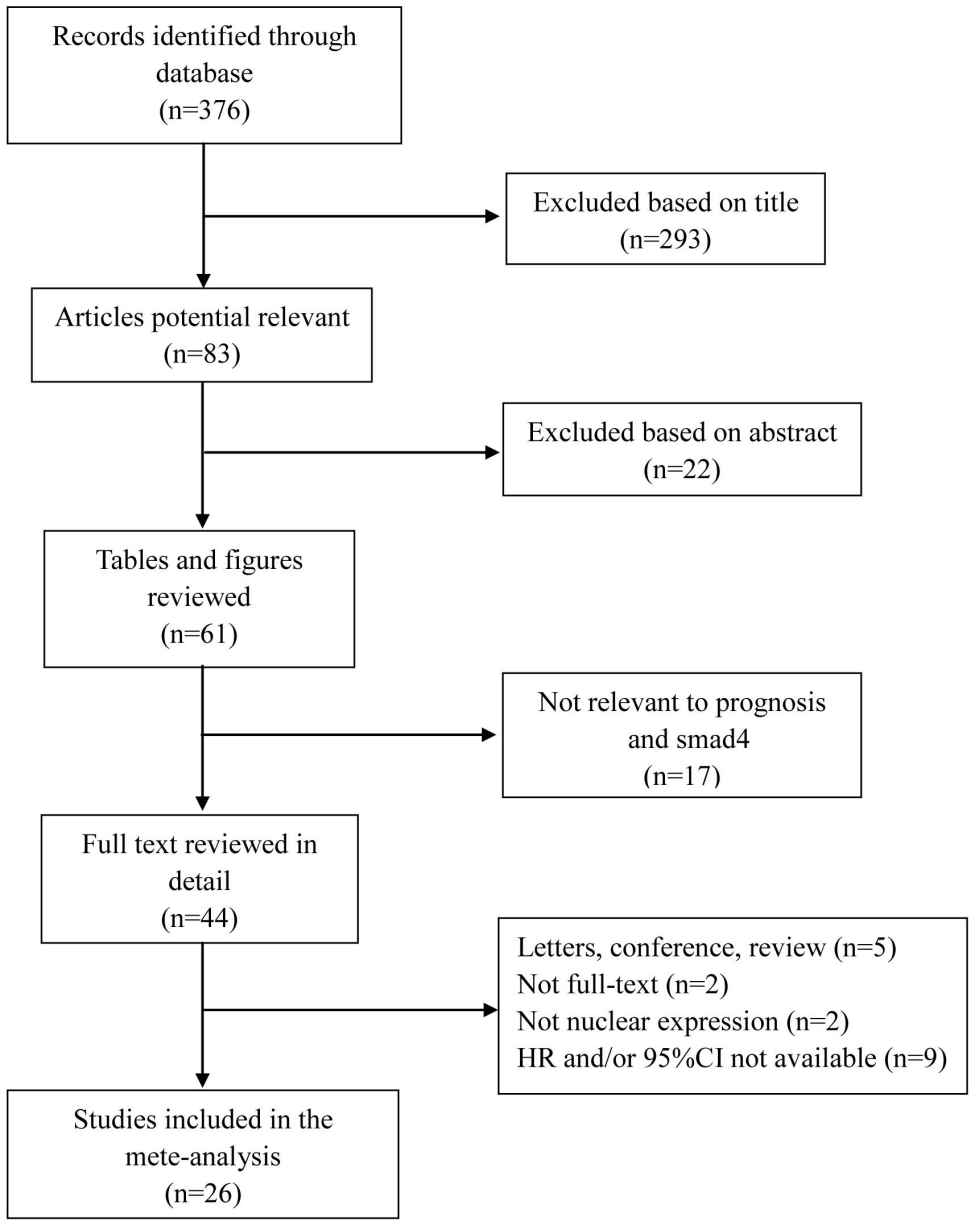
Methodological flow diagram of the meta-analysis.

A total of 376 articles were identified by the initial search. Figure 1 shows the detailed screening process. After careful screening of the titles, abstracts, figures and key data, 26 articles were included in our meta-analysis according to the inclusion criteria [11], [12], [17]–[40]. Only two articles evaluated the prognostic value for RFS, and six evaluated the prognostic value for CSS. Considering that the number of studies for these two indicators was small, we combined the data for CSS with RFS. Thus, 20 studies involving 4247 patients and evaluating OS and 8 studies involving 3323 cases for RFS/CSS were analyzed in our meta-analysis. As shown in Table 1, the ethnicity background of patients was classified as Caucasian or Asian. The number of patients ranged from 34 to 1404. The patients were diagnosed with various carcinomas, among which digestive tumors accounted for most carcinomas, particularly pancreatic cancer (n = 10), colorectal cancer (n = 7) and gastric cancer (n = 4). And the remaining 5 studies included other tumor types.

**Table 1:** Main characteristics of the studies included in this meta-analysis.

Author	Year	Country	Ethnicity	Type	Stage	Number	Follow up (months) (median and/or range)
Milind Javle	2014	Greece	Caucasian	Pancreatic	NA	81	NA
Jeong Hwan Park	2013	Korea	Asian	Renal	I–IV	637	74 (2–187)
Minoru Oshima	2013	Japan	Asian	Pancreatic	I–II	106	17.3 (2.8–124.8)
Arnaud D. Roth	2012	Switzerland	Caucasian	Colorectal	II–III	1404	69
Adriana Handra-Luca	2012	France	Caucasian	Pancreatic	NA	99	26 (1.2–95.5)
J. B. Bachet	2011	France	Caucasian	Pancreatic	I–II	444	54 (1.1–143.7)
Niki A. Ottenhof	2012	Netherlands	Caucasian	Pancreatic	I–III	78	NA
Pavlos Lampropoulos	2012	Greece	Caucasian	Colorectal	I–IV	195	56 (1–72)
Martin Isaksson	2011	Sweden	Caucasian	Colorectal	I–IV	441	NA
Philip W. Voorneveld	2012	Netherlands	Caucasian	Colorectal	NA	209	65 (1.5–257)
Maartje G. Noordhuis	2011	Netherlands	Caucasian	Cervical	I–II	255	66 (4–223)
Xuemei Li	2011	China	Asian	Colorectal	NA	147	NA
Hirokazu Okano	2004	Japan	Asian	Gastric	NA	166	48.8(1.8–136.2)
By Andrew V. Biankin	2002	Australia	Caucasian	Pancreatic	NA	114	3.5 (0–117)
Kyu Yeoun Won	2009	Korea	Asian	Osteosarcoma	I–IV	34	NA
Judith N Kloth	2008	Netherlands	Caucasian	Cervical	NA	117	NA
Li-HuiWang	2007	Korea	Asian	Gastric	I–IV	114	NA
Mesker We	2009	Netherlands	Caucasian	Colorectal	I–II	135	NA
Che Xiangming	2001	Japan	Asian	Gastric	NA	249	73.5 (54–76)
Y. H. Kim	2003	USA	Caucasian	Gastric	I–IV	304	67 (1–72)
Martin Isaksson-Mettavainio	2006	Sweden	Caucasian	Colorectal	NA	86	NA
Kiyokazu Hiwatashi	2009	Japan	Asian	Hepatocellular	I–IV	121	53
Metin Tascilar	2001	USA	Caucasian	Pancreatic	I–IV	249	17
Khorana AA	2005	USA	Caucasian	Pancreatic	NA	124	NA
Zhan Hua	2003	China	Asian	Pancreatic	I–IV	34	NA
Tomoko Toga	2004	Japan	Asian	Pancreatic	I–IV	88	NA

NA: not available.

### Main results

**Figure 2:**
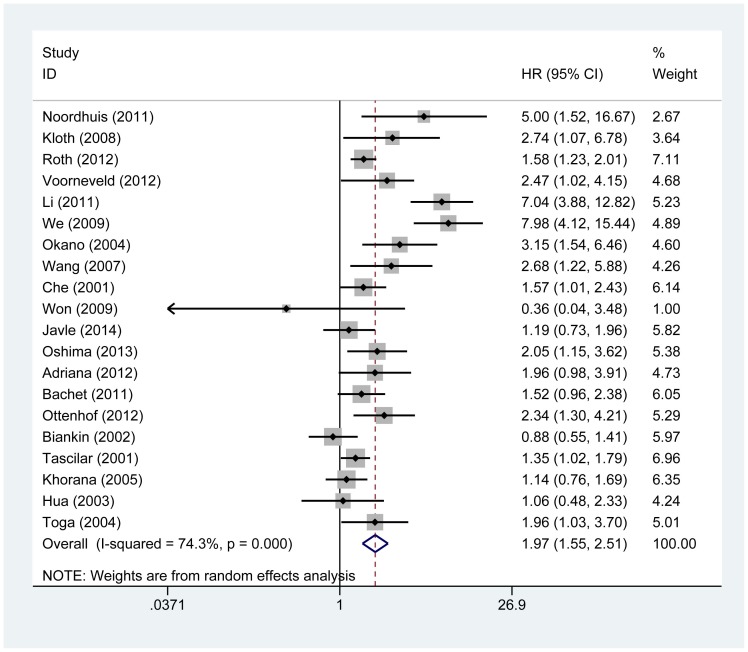
Forest plots of studies evaluating hazard ratios (HRs) of Smad4 for overall survival.

As shown in Table 2, we found that loss of Smad4 staining predicted a poor outcome with a pooled HR of 1.97 (95% CI: 1.55–2.51; P_heterogeneity_<0.001) for 20 studies evaluating OS (Figure 2). Similarly, the prognostic role of Smad4-negative expression for RFS/CSS was also investigated with a combined HR of 1.81 (95% CI: 1.30–2.54; P_heterogeneity_<0.001).

**Table 2:** Meta-analysis results.

Outcome	Variables	Number of studies	Model	HR(95%CI)	Pheterogeneity
OS	ALL	20		1.97 (1.55–2.51)	<0.001
	Cancer type				
	Cervical	2	Fixed	3.43 (1.65–7.12)	0.436
	Colorectal	4	Random	3.76 (1.53–9.25)	<0.001
	Gastric	3	Fixed	2.02 (1.44–2.83)	0.197
	Pancreatic	9	Fixed	1.39 (1.19–1.61)	0.185
	Ethnicity				
	Asian	7	Random	2.24 (1.32–3.81)	<0.001
	Caucasian	13	Random	1.82 (1.39–2.37)	<0.001
	Participate number				
	Large	6	Fixed	1.56 (1.34–1.82)	0.277
	Small	14	Random	2.09 (1.42–3.06)	<0.001
CSS/RFS	ALL	8		1.81 (1.30–2.54)	<0.001
	Cancer type				
	Colorectal	5	Random	2.54 (1.46–4.39)	<0.001
	Others	3	Fixed	1.24 (0.95–1.61)	0.544
	Ethnicity				
	Asian	2	Fixed	1.34 (0.99–1.81)	0.647
	Caucasian	6	Random	2.12 (1.32–3.43)	<0.001
	Participate number				
	Large	4	Fixed	1.41 (1.19–1.67)	0.353
	Small	4	Random	2.89 (1.34–6.24)	<0.001

OS: overall survival; CSS: cancer-specific survival; RFS: recurrence-free survival; HR: hazard ratio; CI: confidence interval; Small: studies with less than 200 participants; Large: studies with more than 200 participants.

Regarding OS, subgroup analyses by cancer type showed that loss of Smad4 staining predicted an unfavorable prognosis in cervical carcinoma (HR = 3.43, 95% CI: 1.65–7.12; P_heterogeneity_ = 0.436), gastric cancer (HR = 2.02, 95% CI: 1.44–2.83; P_heterogeneity_ = 0.197), colorectal cancer (HR = 3.76, 95% CI: 1.53–9.25; P_heterogeneity_<0.001) and pancreatic cancer (HR = 1.39, 95% CI: 1.19–1.61; P_heterogeneity_ = 0.185). When ethnicity was considered, results revealed that negative Smad4 expression indicating poor OS was found both in Asian cases (HR = 2.24, 95% CI: 1.32–3.81; P_heterogeneity_<0.001) and Caucasian populations (HR = 1.82, 95% CI: 1.39–2.37; P_heterogeneity_<0.001). Subgroup analysis stratified by the number of participants (Since mean number of participants was close to 200, studies with more than 200 participants were classified as “large” and studies with less than 200 patients were classified as “small”) revealed that inactivation of Smad4 was closely correlated with poor OS regardless of the number of patients (Large: HR = 1.56, 95% CI: 1.34–1.82; P_heterogeneity_ = 0.277; Small: HR = 2.09, 95% CI: 1.42–3.06; P_heterogeneity_<0.001).

Regarding RFS/CSS, when different ethnicities were considered, Smad4-negative expression was a negative prognostic marker for Caucasian patients (HR = 2.12, 95% CI: 1.32–3.43; P_heterogeneity_<0.001) than for Asian patients (HR = 1.34, 95% CI: 0.99–1.81; P_heterogeneity_ = 0.647). Subgroup analyses by cancer type showed that loss of Smad4 staining was associated with a worse outcome in colorectal cancer (HR = 2.54, 95% CI: 1.46–4.39; P_heterogeneity_<0.001). When performing subgroup analyses stratified by the number of participants, we found that inactivation of Smad4 predicted worse survival regardless of the number of participants (Large: HR = 1.41, 95% CI: 1.19–1.67; P_heterogeneity_ = 0.353; Small: HR = 2.89, 95% CI: 1.34–6.24; P_heterogeneity_<0.001).

**Figure 3:**
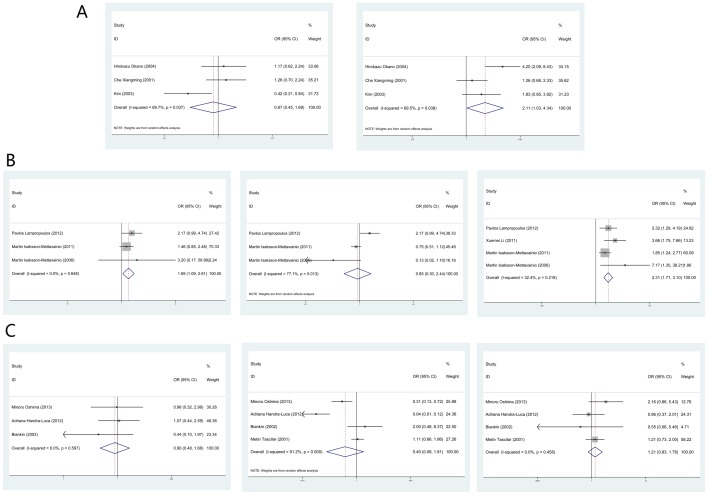
Forest plots of studies evaluating the association between Smad4 and clinical parameters. In gastric cancer (A): histology (left; differentiated vs. undifferentiated); lymph node (right; absent vs. present). In colorectal cancer (B): age (left; old vs. young); gender (middle; male vs. female); TNM stage (right; advanced vs. early). In pancreatic cancer (C): differentiation (left; well/moderate vs. poor); lymph node (middle; absent vs. present); tumor size (right; small vs. large).

The relationship between clinical parameters (reported in more than 2 studies) and Smad4 staining was explored in gastric cancer, colorectal cancer and pancreatic cancer (Figure 3). In gastric cancer, loss of Smad4 staining was found to be significantly associated with the rate of lymph node metastasis (OR = 2.11, 95% CI: 1.03–4.34; P_heterogeneity_ = 0.038) but not with tumor histology (OR = 0.87, 95% CI: 0.45–1.69; P_heterogeneity_ = 0.037). When colorectal cancer was analyzed, age (OR = 1.69, 95% CI: 1.09–2.61; P_heterogeneity_ = 0.648) and stage (OR = 2.31, 95% CI: 1.71–3.10; P_heterogeneity_ = 0.218) but not gender (OR = 0.85, 95% CI: 0.30–2.44; P_heterogeneity_ = 0.013) were positively associated with loss of Smad4 staining. However, no significant relationship was found between Smad4 staining and clinical parameters in pancreatic cancer, including tumor differentiation (OR = 0.90, 95%CI: 0.48–1.68; P_heterogeneity_ = 0.597), lymph node metastasis (OR = 0.40, 95%CI: 0.09–1.81; P_heterogeneity_<0.001) or tumor size (OR = 1.21, 95%CI: 0.83–1.78; P_heterogeneity_ = 0.458).

### Heterogeneity

To explore the potential source of heterogeneity among studies, a meta-regression was conducted that utilized the following variables: year of publication, ethnicity, cancer type and number of participants (large vs. small). For OS, the results showed that year of publication, ethnicity and number of participants did not contribute to the source of heterogeneity. Cancer type was an exception (p = 0.003), and this variable could explain 46.36% of the heterogeneity. For RFS/CSS, no variable included in the meta-regression appeared to be a source of heterogeneity.

### Sensitivity analysis

**Figure 4:**
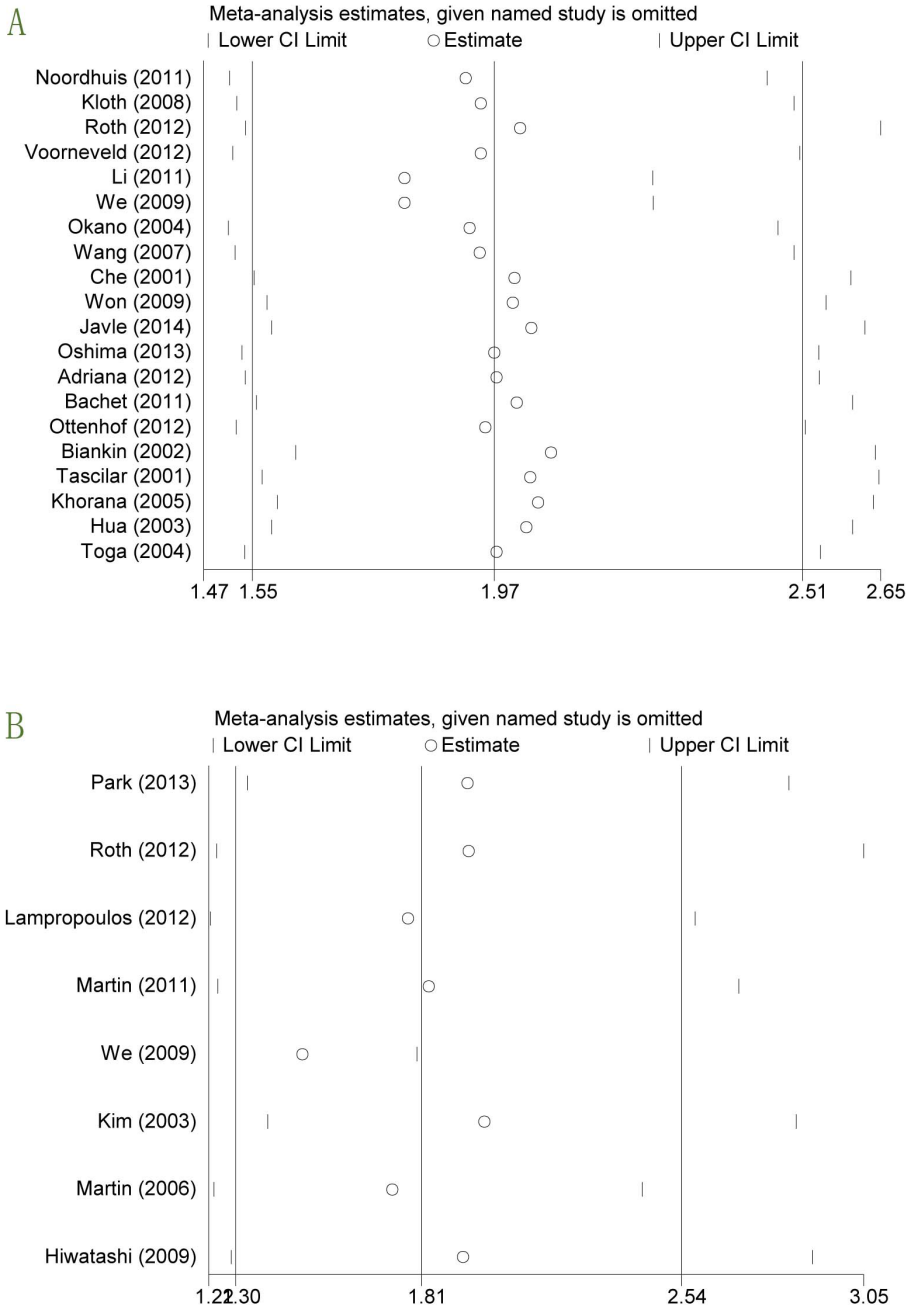
Effect of individual studies on the pooled hazard ratio (HR) for OS (A) and RFS/CSS (B).

We sequentially removed studies to investigate the influence of an individual study on the pooled results. As shown in Figure 4, pooled HRs were found to be stable, suggesting that no individual study significantly affected the pooled results.

### Publication bias

**Figure 5:**
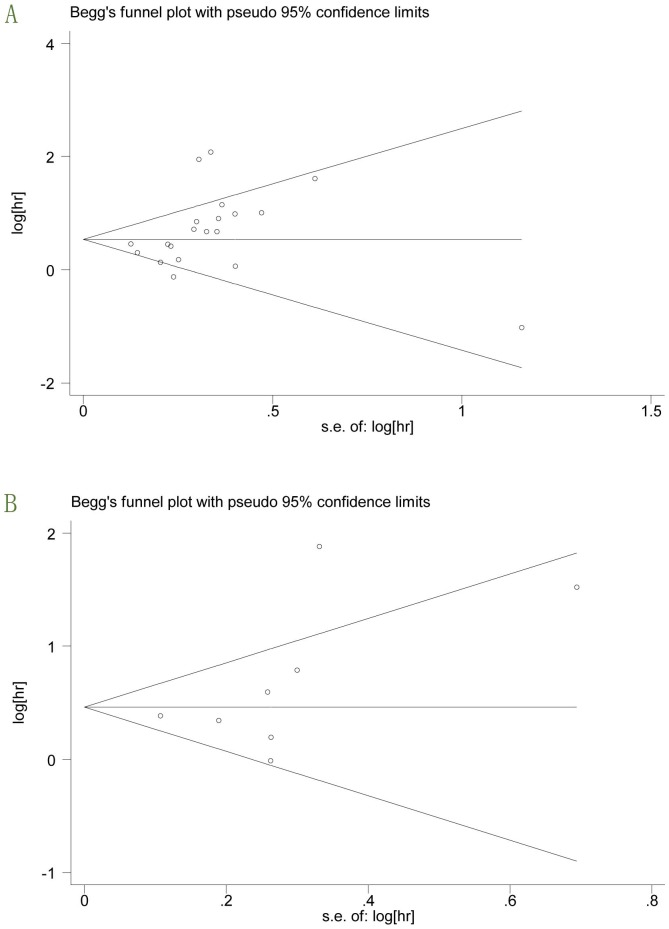
Begg's funnel plots for all of the included studies reported with OS (A) and RFS/CSS (B).

Begg's funnel plot and Egger's linear regression test were used to assess publication bias. Begg's funnel plot for both OS (P = 0.05) and RFS/CSS (P = 0.108) revealed no obvious bias. Further confirmation using Egger's regression test also failed to find evidence of a publication bias for OS (P = 0.088) and RFS/CSS (P = 0.229). There was no evidence of a significant publication bias in the meta-analysis because the P values were not <0.05 (Figure 5).

## Discussion

Cancer is a major public health problem worldwide and is a complex process resulting from environmental factors, genetics, and their interactions. Due to the lack of an early diagnosis and of effective treatment for various cancers, the prognosis of a cancer patient is often poor. Various predictors such as TNM stage, genetic factors, and inflammatory factors have been identified and applied for determining the prognosis of patients with various carcinomas. Some proteins that could act as tumor suppressors or oncogenes, such as p53 and EGFR, have also been reported to be effective biomarkers for providing a prognosis [41]–[43]. Smad4, a tumor suppressor gene located on chromosome 18q21.1, was recently reported to predict clinical outcomes in some cancers. However, the prognostic value of this marker in various cancers has remained inconclusive. Thus, we conducted a meta-analysis to evaluate the prognostic role of Smad4 in various cancers.

In our meta-analysis, which includes 26 studies involving 7570 patients, it is interesting to note that loss of Smad4 staining was strongly associated with a worse prognosis for OS and CSS/RFS. Subgroup analyses revealed that unfavorable OS with Smad4-negative expression could be found in both Asian and Caucasian cases. Additionally, loss of Smad4 staining was a significant prognostic marker for a poor outcome in different cancers (cervical carcinoma, colorectal cancer, gastric cancer and pancreatic cancer), regardless of the number of participants (small or large). Compared with the previous meta-analysis, the present study includes more studies and individuals and thus might produce a more comprehensive result than the study of RA et al. [44] that identified vascular endothelial growth factor (VEGF), bcl2, p16 and bax but not Smad4 as immunohistochemical prognostic markers in pancreatic cancer. Worse CSS/RFS with negative Smad4 could be found in Caucasian populations but not in Asian cases. Subgroup analyses revealed that unfavorable CSS/RFS with loss of Smad4 staining was also found in colorectal cancer, regardless of the number of participants. Meta-regression was performed to investigate the potential source of heterogeneity, and cancer type was found to explain most of the heterogeneity for studies evaluating OS in our meta-analysis. However, this type of heterogeneity is difficult to exclude because the recruitment of sufficient patients with a specific type of cancer is difficult. Future studies including more cancer types and a larger number of participants are needed to explore the prognostic value of Smad4 in specific cancers.

As a member of the Smad family, Smad4 plays an important role in the transforming growth factor β (TGF-β) signaling pathway from the cell surface to the nucleus. Activation of Smad4 under different conditions may result in apoptosis or growth arrest in the G1 phase of the cell, responses that are primarily associated with the development of several tumors [45], [46]. In addition, the inactivation of the Smad4 gene within an evolving neoplasm may indirectly influence the extracellular matrix to promote neoplastic growth and affect the prognosis. In our study, loss of Smad4 staining expression was associated with unfavorable outcomes for various cancers, a finding that was consistent with the results of previous studies. We also explored the association between Smad4 expression and clinico-pathological factors. In gastric cancer, patients with Smad4-nagative expression had high rates of lymph node involvement. It was interesting to determine that inactivation of Smad4 in patients with colon cancer was more likely to be found in older populations and in patients with a later tumor stage. However, in pancreatic cancer, no obvious relationship was found between Smad4 staining and clinical parameters. The results might suggest that the exact biology or mechanism of SMAD4 is likely different for divergent tumor types. Thus, more studies that include more cancer types are needed to assess the association of clinical parameters and Smad4 and explore the appropriate mechanisms in the future.

Some limitations exist regarding this meta-analysis. First, most of the enrolled studies were retrospective, making them more susceptible to some bias. Second, all of the included studies measured Smad4 expression by immunohistochemistry. Many factors, such as the primary antibody and antibody concentration, may affect the results. However, it was impossible to perform a subgroup analysis to investigate the potential effect of the technique on the combined result. Third, heterogeneity among studies due to cancer type was relative large in our meta-analysis. Many other factors may also contribute to heterogeneity. However, due to the lack of sufficient information, our analysis was limited. More related analyses are needed in the future. Moreover, although we tried to identify as many pertinent studies as possible, limited databases were searched, which might reduce the persuasive power of the pooled estimate to some extent.

In summary, this study demonstrated that loss of Smad4 staining was a poor predictor for survival in patients with various cancers. To make better use of Smad4 and apply the potential prognostic factor clinically, additional studies are needed to provide a more detailed picture of the clinical relevance and biological mechanism of Smad4.

## Supporting Information

Checklist S1 PRISMA checklist.
(DOC)
